# Spermine-Related DNA Hypermethylation and Elevated Expression of Genes for Collagen Formation are Susceptible Factors for Chemotherapy-Induced Hand-Foot Syndrome in Chinese Colorectal Cancer Patients

**DOI:** 10.3389/fphar.2021.746910

**Published:** 2021-09-01

**Authors:** Mingming Li, Jiani Chen, Shaoqun Liu, Xiaomeng Sun, Huilin Xu, Qianmin Gao, Xintao Chen, Chaowen Xi, Doudou Huang, Yi Deng, Feng Zhang, Shouhong Gao, Shi Qiu, Xia Tao, Jingwen Zhai, Hua Wei, Houshan Yao, Wansheng Chen

**Affiliations:** ^1^ Department of Pharmacy, Second Affiliated Hospital of Naval Medical University, Shanghai, China; ^2^ Department of Gastric Intestinal Surgery, Minhang Hospital, Fudan University, Shanghai, China; ^3^ Research Institute, GloriousMed Clinical Laboratory Co., Ltd., Shanghai, China; ^4^ Institutes of Biomedical Sciences, Fudan University, Shanghai, China; ^5^ Department of General Surgery, Second Affiliated Hospital of Naval Medical University, Shanghai, China; ^6^ Traditional Chinese Medicine Resource and Technology Center, Shanghai University of Traditional Chinese Medicine, Shanghai, China; ^7^ Department of Pharmacy, 905th Hospital of PLA Navy, Naval Medical University, Shanghai, China

**Keywords:** adverse effects, chemotherapy, DNA methylation, hand-foot syndrome, immune response, multi-omics, spermine, susceptible factors

## Abstract

Hand-foot syndrome (HFS) is a common capecitabine-based chemotherapy-related adverse event (CRAE) in patients with colorectal cancer (CRC). It is of great significance to comprehensively identify susceptible factors for HFS, and further to elucidate the biomolecular mechanism of HFS susceptibility. We performed an untargeted multi-omics analysis integrating DNA methylation, transcriptome, and metabolome data of 63 Chinese CRC patients who had complete CRAE records during capecitabine-based chemotherapy. We found that the metabolome changes for each of matched plasma, urine, and normal colorectal tissue (CRT) in relation to HFS were characterized by chronic tissue damage, which was indicated by reduced nucleotide salvage, elevated spermine level, and increased production of endogenous cytotoxic metabolites. HFS-related transcriptome changes of CRT showed an overall suppressed inflammation profile but increased M2 macrophage polarization. HFS-related DNA methylation of CRT presented gene-specific hypermethylation on genes mainly for collagen formation. The hypermethylation was accumulated in the opensea and shore regions, which elicited a positive effect on gene expression. Additionally, we developed and validated models combining relevant biomarkers showing reasonably good discrimination performance with the area under the receiver operating characteristic curve values from 0.833 to 0.955. Our results demonstrated that the multi-omics variations associated with a profibrotic phenotype were closely related to HFS susceptibility. HFS-related biomolecular variations in CRT contributed more to the relevant biomolecular mechanism of HFS than in plasma and urine. Spermine-related DNA hypermethylation and elevated expression of genes for collagen formation were closely associated with HFS susceptibility. These findings provided new insights into the susceptible factors for chemotherapy-induced HFS, which can promote the implementation of individualized treatment against HFS.

## Introduction

According to the National Comprehensive Cancer Network (NCCN) guidelines and routine clinical practice, the capecitabine-based chemotherapy XELOX (capecitabine plus oxaliplatin) is the most recommended and adopted treatment (Benson et al., 2018) for types of solid tumors, including colorectal cancer (CRC) (Bray et al., 2018). Owing to the cytotoxicity of capecitabine, however, XELOX can cause various chemotherapy-related adverse events (CRAEs), one of the most common of which is **the** hand-foot syndrome (HFS, also called palmar-plantar erythrodysesthesia). It ranks within the top 3 CRAEs with an incidence rate of up to 70% (Lou et al., 2016; Yap et al., 2017; Deng et al., 2020; Li et al., 2021). HFS symptoms include redness, swelling, as well as pain on the palms of the hands and the soles of the feet. Consequently, HFS can influence a patient’s adherence to a chemotherapy regimen and adversely affect his/her quality of life.

Personalized medicine can help prevent CRAEs because a personalized approach ensures the proper selection of drugs and their dosages. One of the prerequisites for personalized medicine is predictive markers. However, the CRAE biomarkers need to be optimized in at least two aspects. First, the currently available HFS biomarkers focus mainly on capecitabine metabolism. Germline polymorphisms of genes controlling drug metabolism (Lam et al., 2016) and each drug’s pharmacokinetic parameters (Daher Abdi et al., 2014) are related to HFS. Nonetheless, the response to a particular cytotoxic substance (including a drug) may vary amongst individuals, which can also contribute to the sensitivity to CRAEs. Notably, our preliminary study has revealed potential valuable CRAE biomarkers derived from both endogenous urine metabolites (Deng et al., 2020) and germline DNA methylation (M et al., 2021). Second, the pathological mechanism of HFS requires further investigation. Although the most widely accepted mechanism involves inflammation mediated by cyclooxygenase 2 (*COX2*) overexpression (Lou et al., 2016), a prospective study reported that pyridoxine, which suppresses inflammation, cannot effectively prevent HFS (Yap et al., 2017). Our limited understanding of the HFS mechanism may be partially attributable to the lack of any apparent linkage between HFS-related alterations in cell biochemistry as assessed with multi-omics datasets.

To further address the mechanism underlying HFS, we conducted an integrated multi-omics analysis of 63 CRC patients before they received adjuvant chemotherapy. The analysis integrated matched multi-omics data for normal colorectal tissue (CRT), including transcriptome and DNA methylation, plus the metabolome data for CRT, plasma, and urine samples. Dietary intake data were also recorded during flow-up. The results advance our understanding of HFS biochemistry and provide better biomarkers, which can further facilitate individualized treatment against HFS.

## Materials and Methods

### Patient Selection and Sample Collection

**FIGURE 1:**
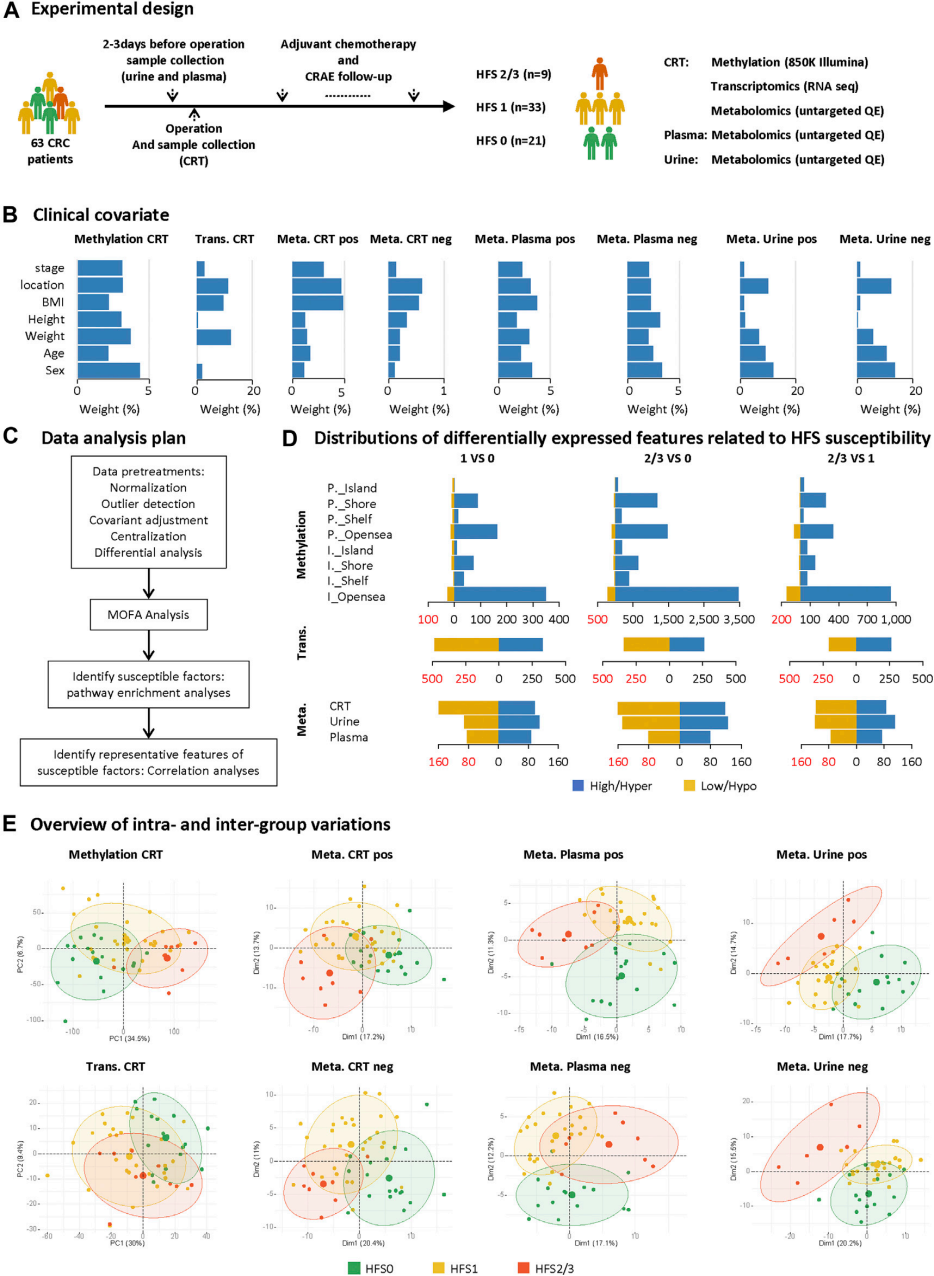
Study design and differential analyses on multi-omics datasets. **(A)** Sample collection and the experimental design. **(B)** The weighted average proportion variance of each clinical covariate effect in each omics dataset (CRT methylation, CRT transcriptome, CRT/plasma/urine metabolome). **(C)** Data analysis plan. **(D)** Distributions of differentially expressed features (DEFs) related to HFS susceptibility in each omics dataset. **(E)** Overview of intra- and inter-group variations. A principal components analysis (PCA) plot was generated using DEFs from each omics dataset. The metabolome data were acquired under positive (pos) and negative (neg) modes and are plotted separately. The circle of each group represents the 75% confidence interval. Abbreviations: CRC, colorectal cancer; CRAE, chemotherapy-related adverse event; CRT, normal colorectal tissue; DEF, differentially expressed feature; Meta, metabolome; Trans, transcriptome; P._Island, promoter-island region; P._Shore, promoter-shore region; P._Shelf, promoter-shelf region; P._Opensea, promoter-opensea region. Here, we focused on DMPs mapped to a specific gene, and thus DMPs in intergenic regions are not displayed here.

Figure 1A illustrates the study design. Patients were selected from a registered ongoing clinical trial (registered at www.clinicaltrials.gov, NCT03030508) carried out at Shanghai Changzheng Hospital from January 2018 to April 2019. The study protocol was approved by the Biomedical Research Ethics Committee of Shanghai Changzheng Hospital, and written consent was obtained from each patient. Inclusion criteria were: 1) over 18 years old; 2) CRC confirmed by biopsy; 3) the first treatments were resection followed by capecitabine-based adjuvant chemotherapy. CRAEs were recorded according to Common Terminology Criteria for Adverse Events (CTCAE, Version 4.0), based on which patients were divided into three groups: grade 0 HFS (HFS0), grade 1 HFS (HFS1), and grade 2/3 HFS (HFS2/3) (**Additional file 2:** Supplementary Table S1).

Fasting urine samples were collected into 15-ml Falcon tubes, and plasma samples were collected into 15-ml EDTA tubes 1–3 days before resection. CRT was collected during resection. Immediately after resection, samples were taken from the CRC tumor and the adjacent CRT (approximately 5–10 cm away from the tumor site), and the entire CRT was then separated from the tumor. All samples were stored at −80°C for later analysis. A total of 63 cases with stored frozen tissue samples and complete CRAE records were selected for subsequent analyses, which included 21 grade 0 HFS, 33 grade 1 HFS, and 9 grade 2/3 HFS patients.

### Metabolome Assays

The metabolome assays were completed on a UHPLC system coupled to a quadruple time-of-flight mass spectrometer. Each prepared sample was screened under both positive and negative ionization modes (see the detailed descriptions of these methods in Additional file 1: Supplementary Material). Metabolite data were processed through multiple steps, including 1) best-matched internal standard correction for peak areas, with an additional creatinine correction for the urine metabolome, 2) 80% filtering rule and cutoff for >40% coefficient of variation, outlier detection, and imputation, and 3) normalization and transformation: quantile normalization, log10 transformation, Pareto scaling (Bijlsma et al., 2006).

### DNA Methylation Assays

DNA was extracted from frozen tissue samples using the Takara Genomic DNA Purification Kit. DNA was quantified using a Qubit 2.0 fluorometer (Invitrogen, Waltham, MA, United States). Bisulfite conversion of DNA was conducted using the Zymo bisulfite D5005 kit. Microarray assays were performed according to Illumina’s standard protocol with the EGMK91396 kit. Processed methylation chips were scanned using an iScan reader (Illumina, San Diego, CA, United States).

Methylation data were analyzed using the ChAMP package (Tian et al., 2017). The extent to which each probe was methylated was calculated as the β-value: β = intensity (methylated) ÷ intensity (methylated + unmethylated). Probes were accepted if they had significantly higher intensity (*p* ≤ 0.05, compared with negative controls on each chip) and higher frequency (≥95% amongst all samples). Exclusion of probes from non-CpG sites, sex chromosomes, and those containing a known SNP(s) yielded 753,722 probes that were subsequently normalized with respect to beta-mixture quantile, and probes having a coefficient of variation value of >20% were excluded.

### RNA Sequencing

RNA sequencing was performed on 54 normal CRT samples. Libraries were prepared using the Illumina TruSeq stranded mRNA sample preparation kit, starting with 2 μg RNA. Oligo(dT) magnetic beads were used to reduce the abundance of ribosomal RNA. The ribosomal RNA–depleted RNA samples were then randomly cleaved into ∼200-bp fragments, where were then reverse transcribed into strand-specific complementary DNA (cDNA) using random primers. Second-strand cDNA was then synthesized, with dTTP being replaced by dUTP. The resulting double-stranded cDNAs were purified using AMPure XP beads and then subjected to end repair and A-tailing. Finally, each cDNA was ligated to an index adaptor. The products then underwent PCR amplification (15 cycles) using an Illumina cBot system to create the final cDNA libraries that were competent for cluster generation and sequencing using the Illumina NovaSeq 6,000 platform with a paired-end protocol.

Adapter sequences were removed from reads, and low-quality 3′-end fragments were excluded using Skewer (v0.2.2) (Jiang et al., 2014). After quality control using FastQC (v0.11.5, http://www.bioinformatics.babraham.ac.uk/projects/fastqc/), the reads were mapped to reference genome GRCh38 using STAR (v2.5.3a) (Dobin et al., 2013) with default parameters (Dobin and Gingeras, 2015). Software RseQC (v2.6.4) (Wang et al., 2012) was used to check alignment quality, including total mapped reads, reads mapped ratio, and the number of uniquely mapped reads. Read counts for each sample were calculated using HTseq (Anders et al., 2015). The FPKM value, i.e., fragments per kilobase of transcript per million fragments mapped, was calculated using StringTie (v1.3.1c) (Pertea et al., 2016). Genes for which FPKM was >0 in ≥50% of samples were retrained, and outlier detection and imputation were employed, and the log2(FPKM + 1) transformation was used for further analysis.

### Assessment of the Nutritional Status of Patients

To evaluate the contribution of dietary intake to any observed variations in metabolite levels, a simple food-frequency questionnaire (FFQ25) for Shanghai residents was administered to each of the enrolled patients during chemotherapy (Gao et al., 2011).

### Integrated Analysis of Multi-Omics Datasets

We carried out a multi-omics factor analysis (MOFA) (v1.2.0) by integrating differentially expressed features (DEFs) of five omics datasets for CRT DNA methylation, CRT transcriptome, and metabolomes (in both positive and negative modes) from CRT, plasma, and urine with partially shared samples. The following parameters were used: 1) #tolerance, 0.01; 2) #DropFactorThreshold, 0.02; and 3) other parameters were set to default (Argelaguet et al., 2018). Based on the weighted DEFs determined by MOFA, the DEFs at different developmental stages of HFS were further studied by pathway enrichment analyses using the Reactome Database (online available: https://reactome.org/) (Fabregat et al., 2016).

### Construction and Validation of Potential Marker Systems for Hand-Foot Syndrome Prediction

To develop and evaluate the performance of the HFS prediction model based on each omics dataset (except for the transcriptome) separately, we randomly divided the samples into training and validation sets (7:3). While developing the model, a univariate logistic analysis was performed using features with consistent change in both the HFS1 and HFS2/3 groups compared with the HFS0 group to identify features significantly associated with HFS. To further narrow the candidate metabolome marker lists, LASSO (least absolute shrinkage and selection operator) analysis with 10-fold cross-validation and randomForest analysis with 1,000 trees were performed. Based on the intersection of the top 10 features according to the non-zero coefficients in LASSO and mean decrease of accuracy in randomForest, these important HFS-related features were combined to establish a HFS prediction model for the multiple combined-marker systems using multivariate logistic regression. To further narrow down DNA methylation makers, a slightly different route was applied. The randomly sampling, LASSO-logistic and randomForest modeling was repeated 5,000 times, and then the fibrosis-related DMPs from the top10 most frequently selected DMP markers were enrolled for DNA methylation model construction. At last, by using our preliminary data as an independent validation dataset, which contained DNA methylation data from 21 CRC patients (M et al., 2021) (https://www.ncbi.nlm.nih.gov/geo/query/acc.cgi?acc=GSE149282), we tested our DNA methylation model. Unfortunately, so far there is no other public dataset available for further validation. ROC curves were analyzed to evaluate the predictive performance of each model. A flow chart for the model development and validation is presented in Figure 8A.

### Statistical Analysis

We eliminated the effects of baseline and potential confounding factors of sex, age, weight, height, body mass index (BMI), CRC location, and CRC stage (Combat method in ChAMP for methylation, removeBatchEffect method in limma for all others). Differential expression analyses were performed to identify HFS-related features in each omics dataset (ChAMP for methylation and limma for transcriptome/ metabolome data) (Ritchie et al., 2015; Tian et al., 2017). DEFs for the methylation, the transcriptome, and the metabolome were filtered based on the following respective criteria: |∆ β-values| > 0.1; |log2 FC| > [mean ± 2 SD]; |log10FC| > 0. The “pvca” package was used for exploring the variation of the covariate effect. The “CIBERSORT” package was employed for the analysis of the abundance of 22 infiltrating immune cells for transcriptome data (Newman et al., 2019). The “glmnet” and “randomForest” packages were respectively applied for LASSO and randomForest analyses to select features. The “pROC” package was used for the analysis and visualization of AUROC. Gephi software (v0.9.2) was used to generate a network based on the results of the correlation analysis (Bastian et al., 2009). All statistical analyses were performed using R software (v3.6.3), and *p* < 0.05 was considered statistically significant.

## Results

### Patient Characteristics

The 63 CRC patients all received capecitabine-based chemotherapy on a 3-weeks cycle after resection surgery. The study design is illustrated in Figure 1A. Patient samples of CRT, plasma, and urine were collected before chemotherapy. During each cycle, patients received oxaliplatin (0.16–2 g/d) intravenously on day one and capecitabine (1.5 g/d) orally for the first two weeks. These patients received chemotherapy for at least three cycles, and the median number of chemotherapy cycles was eight. These multi-omics datasets collected from those patients were affected by several covariates, the variation proportion of which are shown in Figure 1B using principal variance component analysis (PVCA) (Bushel, 2020); and the influence of these covariates were adjusted before the subsequent analysis (Figure 1C).

### Differentially Expressed Features Generated on the Multi-Omics Datasets

To comprehensively explore the mechanism of HFS development from different perspectives, we initially performed differential expression analysis for each omics dataset between any two of the three HFS grades, namely HFS0, HFS1, and HFS2/3 (Figure 1D). The following numbers of DEFs were identified: 15,152 DEFs for methylation of CRT genomic DNA, 1,449 for the CRT transcriptome, 467 for the CRT metabolome, 327 for the plasma metabolome, and 442 for the urine metabolome (**Additional file 2:** Supplementary Table S2). The results indicated that DNA hypermethylation was the most common susceptibility marker for HFS. The number of hypermethylated DMPs correlated positively with HFS severity (Figure 1D). Most of the hypermethylated DMPs were in the CpG-shore and opensea regions. Overall, most HFS-related transcriptome features were downregulated. However, the number of downregulated transcriptome features correlated negatively with HFS severity. For the HFS-related metabolome data, the majority of the metabolites in CRT were downregulated. Principal component analysis (PCA) for each omics dataset revealed that the DEFs could be reasonably categorized into the three HFS grades (Figure 1E). The smallest sum of the first two principal components was 27.8% [plasma metabolome profile (pos)], and the largest sum was 41.2% (CRT genomic DNA methylation profile).

### Integrated Multi-Omics Response Profiles

**FIGURE 2:**
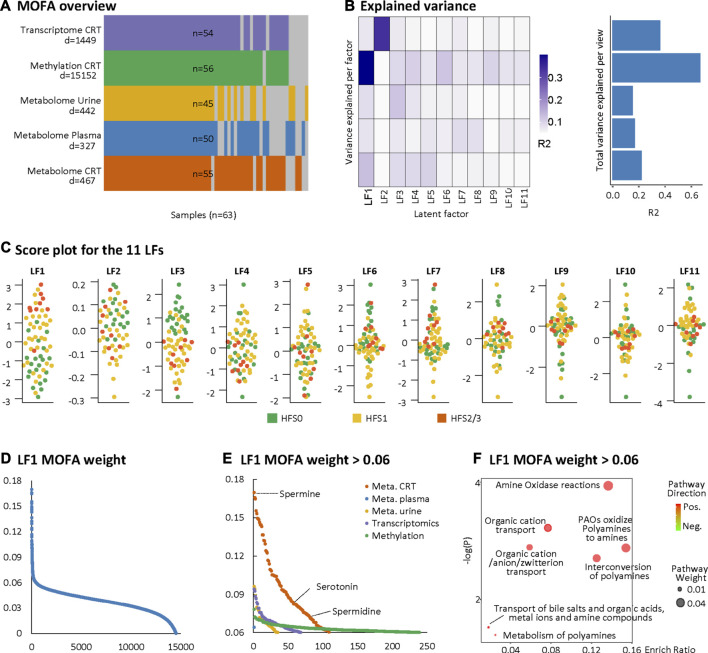
Multi-omics factor analysis (MOFA). MOFA captured 11 latent factors (LFs) from the multi-omics datasets. **(A)** MOFA overview listing the number of samples and differentially expressed features (DEFs) from each omics dataset. **(B)** Explained variance (R2) by each LF (left) and the cumulative proportion of total variance explained (right) for each omics dataset of the MOFA model. **(C)** Score plots for the 11 LFs. The *y*-axis represents the each LF score for each sample. Colors denote sample groups. **(D)** The distribution of features based on the LF1 weight of the MOFA model. **(E)** The distributions of features with LF1 MOFA weight above the threshold of 0.06 from each omics dataset. **(F)** Pathway enrichment analysis of DEFs with LF1 MOFA weight > 0.06 (in HFS2/3), including CRT metabolome and CRT transcriptome. Cutoff: the minimum number of DEFs enriched in the pathway was set as 2, the *p*-value was set as 0.05, and –log10(P) was used for visualization. Pathway weight is defined as: (Enrichment ratio) × (sum of absolute value of DEF MOFA L1 weight in the pathway), and the dot size represents pathway weight. Pathway direction is the median fold change (FC) of DEFs for each dataset in the pathway (red, upregulated; green, downregulated). Abbreviations: LF, latent factor; MOFA, Multi-omics Factor Analysis.

We further adopted MOFA to integrate the DEFs of the multi-omics datasets with partially matched samples in an unsupervised manner (Figure 2A). MOFA can accommodate missing values and provide more rigorous statistics than other statistical tools for multi-omics datasets (Argelaguet et al., 2018). MOFA captured a total of 11 latent factors (LFs). All LFs explained 66.8% of the variation in DNA methylation data, followed by 36.0% in the RNA transcriptome, 23.4% in the CRT metabolome, 16.5% in the plasma metabolome, and 15.1% in the urine metabolome. LF1 explained the greatest percentage of variations across all five datasets and yielded the best categorization of the three HFS grades (Figures 2B,C). According to the MOFA weight given by LF1, the CRT metabolome profile had the greatest number of features having a high value of MOFA weight (Figures 2D,E). Within these top-weighted CRT features, spermine had the highest MOFA weight. Spermine, spermidine, and serotonin were enriched in pathways such as amine oxidase reactions, organic cation transport, and polyamine oxidase reactions (Figure 2F).

### Hand-Foot Syndrome-Related Metabolome in Colorectal Tissue, Plasma, and Urine

**FIGURE 3:**
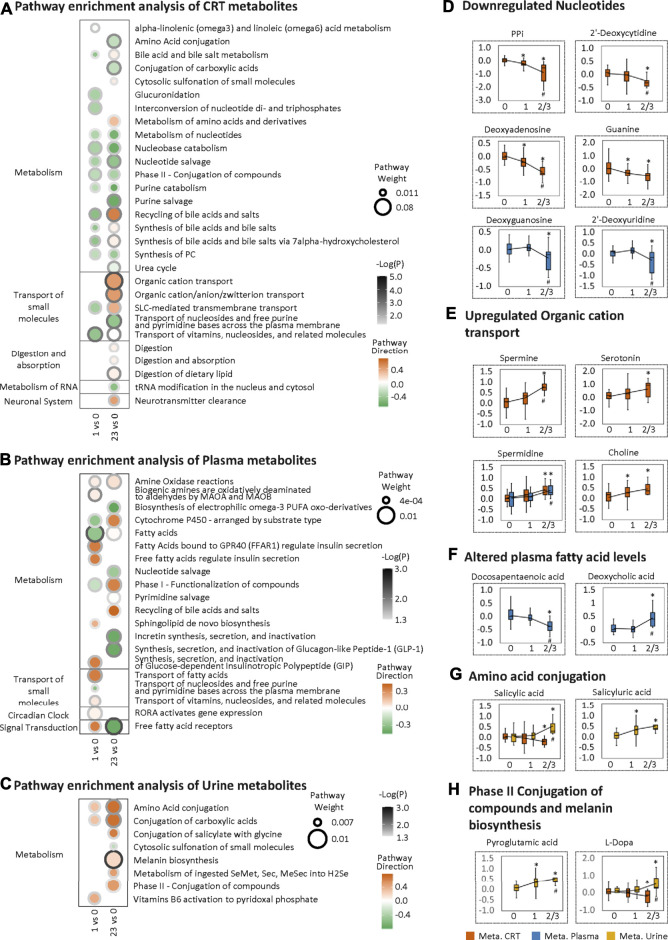
Metabolome changes related to HFS susceptibility. Pathway enrichment analysis of differentially expressed features (DEFs) in CRT metabolome **(A)**, plasma metabolome **(B)**, and urine metabolome **(C)**. Cutoff: the minimum number of DEFs enriched in the pathway was set as 4 **(A)**, 2 **(B)**, and 2 **(C)**, and the *p*-value was set as 0.05. The dot border color represents the –log10(*p*) of pathway significance. Pathway weight is defined as: (Enrichment ratio) × (sum of absolute value of DEF MOFA L1 weight in the pathway), and the dot size represents pathway weight. Pathway direction is the median log10 fold change (FC) of DEFs in the pathway (red, upregulated; green, downregulated). Boxplots in **(D)**, **(E)**, **(F)**, **(G)**, and **(H)** show the relative metabolite levels of the related features enriched in the significantly important pathways in groups HFS0, HFS1, and HFS2/3 (from left to right). Statistical significance was set as: *p <  0.05 for HFS1 vs HFS0, HFS2/3 vs HFS0; ^#^ *p* < 0.05 for HFS2/3 vs HFS1 by differential expression analysis using limma.

To further investigate the LF1 results from the MOFA model, pathway enrichment was analyzed based on MOFA weighted features. For the CRT metabolome, the most significantly altered pathways shared between HFS1 and HFS2/3 were nucleobase catabolism, nucleotide salvage, and nucleotide metabolism, all of which were downregulated in HFS1 and HFS2/3 compared with HFS0 (Figure 3A). Also, the downregulation of nucleotide salvage in HFS2/3 was observed in the plasma metabolome profile (Figure 3B). The development of HFS resulted in decreased levels of free nucleotides and co-factors involved in DNA synthesis in CRT and plasma (Figure 3D). The most significantly enriched pathway was organic cation transport in HFS2/3 (Figure 3A). Several organic cations, including spermine, serotonin, spermidine, and choline, correlated positively with HFS susceptibility (Figure 3E).

For the plasma metabolome, two pathways, namely, those involving fatty acids and free fatty acid receptors, were the most significantly altered pathways, reflecting the characteristics of HFS 1 and HFS2/3, respectively. In these two pathways, docosapentaenoic acid (DPA) had the highest MOFA weight. Amongst all DEFs in the plasma metabolome, deoxycholic acid had the highest MOFA weight (Figure 3F). These findings indicated that a significant change in lipid metabolism in plasma correlated with HFS susceptibility.

For the urine metabolome, the most significantly altered pathways shared between HFS1 and HFS2/3 were amino acid conjugation and conjugation of carboxylic acids (Figure 3C). From DEFs implicated in these pathways, urine salicylic acid and its conjugation product salicyluric acid correlated positively with HFS susceptibility (Figure 3G). Apart from this, HFS2/3 was also characterized by increased melanin biosynthesis. The level of each urine pyroglutamic acid and L-Dopa correlated positively with HFS susceptibility (Figure 3H).

### Spermine, Spermidine, and Serotonin Metabolism and Their Related Metabolic Processes

**FIGURE 4:**
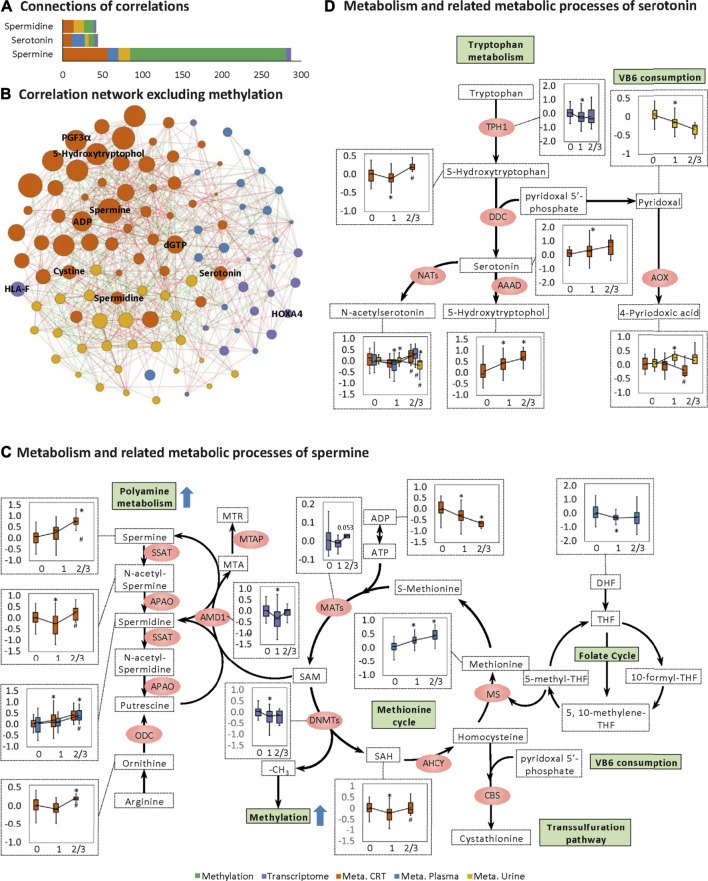
Changes in spermine metabolism related to HFS susceptibility. **(A)** Connections of correlations. The stacked bar charts show the connections between differentially expressed features (DEFs) from all omics datasets with spermine, serotonin, and spermidine (selected based on MOFA LF1 weight and pathway enrichment analysis). **(B)** Correlation network with spermine, serotonin, and spermidine, excluding DNA methylation. A spermine-spermidine-serotonin metabolite-gene correlation network was constructed. The network included only paired DEFs with significant correlations (Spearman correlation, *p* < 0.05) in all omics datasets, excluding DNA methylation. The relatively important DEFs are labeled with feature names. Visualization was achieved with Gephi software (v0.9.2). Node colors represent the type of omics, and node sizes represent the strength of connections between features. Red edges represent positive correlations, and green edges represent negative correlations. **(C, D)** Metabolism and related metabolic processes of spermine **(C)** and serotonin **(D)**. Boxplots show the relative expression levels of the relevant DEFs in groups HFS0, HFS1, and HFS2/3 (from left to right). Statistical significance was set as: *p <  0.05 for HFS1 vs HFS0, HFS2/3 vs HFS0; ^#^ *p* < −0.05 for HFS2/3 vs HFS1 by differential expression analysis using limma.

Notably, for the CRT metabolome, a high MOFA weight was apparent for each of spermine, spermidine, and serotonin involved in organic cation transport (Figures 2E,F). Spermine had the highest MOFA weight amongst all input multi-omics features. Next, correlations between these three features and the rest of the multi-omics features were examined, revealing the strongest connections with methylation, followed by CRT metabolome features, urine metabolome features, plasma metabolome features, and transcriptome features (Figures 4A,B). The metabolomes for the three features and their related metabolic processes were further examined in detail.

Levels of spermine and spermidine increased gradually with the development of HFS severity (Figure 4C). However, the level of their precursor, ornithine, was not elevated in HFS1 but was elevated in HFS2/3 compared with HFS0. The expression level of adenosylmethionine decarboxylase 1 (*AMD1*) was even significantly suppressed in HFS1 but was recovered in HFS2/3 compared with HFS0. The rate-limiting enzyme ornithine decarboxylase (ODC) was not significantly changed in neither the HFS1 nor HFS2/3 group (**Additional file2:** Supplementary Table S3). Moreover, the increase in the fold change (FC) of spermine level in HFS2/3 (compared with HFS0) was greater than that of its precursor ornithine. Therefore, the *de novo* synthesis was not the reason for the elevated spermine level and it may even be repressed as the HFS severity increased. This may lead to the accumulation of SAM. SAM is the methyl-donor for the subsequent methylation. An expected upregulation of SAM may also be contributed by the activated methionine cycle. With the elevated supply of methionine and consumption co-factors ADP, the synthesis of enzyme methionine adenosyltransferase (MAT) had a trend of elevation. Plus, the decreased level of dihydrofolate (DHF) may also contribute to SAM formation through the folate cycle.

The same pattern was also observed for serotonin biosynthesis (Figure 4D). Levels of serotonin and its metabolite 5-hydroxytryptophol were elevated gradually with the increased HFS severity. However, the level of their upstream precursor 5-hydroxytryptophan was significantly suppressed in HFS1 but was recovered in HFS2/3 compared with HFS0 (Figure 4D). Also, the rate-limiting enzyme dopa decarboxylase (DDC) level was not remarkably changed. Therefore, the *de novo* synthesis was not the reason for the elevated serotonin and 5-hydroxytryptophol. In parallel with the upregulation of serotonin and spermine metabolism, the catabolism of the immunosuppressant vitamin B6 (VB6) was also upregulated.

### Spermine, Spermidine, and Serotonin-Related Dietary Intake

**FIGURE 5:**
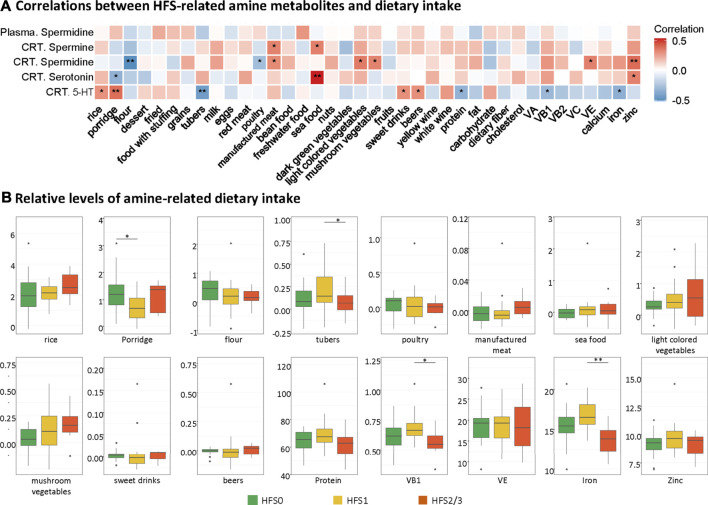
Contribution of dietary intake to the levels of HFS-related amines in CRT. **(A)** Heatmap showing the correlations between HFS-related amine metabolites and dietary intake. Statistical significance was set as: *p <  0.05, **p < −0.01 (Spearman correlation). Red boxes represent positive correlations, and blue boxes represent negative correlations. **(B)** Boxplots show the relative levels of amine-related dietary intake. The Student’s t-test was used to examine the relationship between dietary intake for groups HFS0, HFS1, and HFS2/3, and significance was set as: *p <  0.05, **p < −0.01.

In order to find the possible origin of the difference in the HFS-related amines (namely, spermine, spermidine, and serotonin), we performed the correlation analysis between each amine and each dietary intake (Figure 5A). The intake level of manufactured meat was positively related to both spermine and spermidine in CRT. The intake levels of seafood and zinc positively correlated with both spermidine and serotonin in CRT. Interestingly, although the spermidine levels in plasma were similar to that in CRT, none of the dietary intake was associated with the plasma spermidine levels. Subsequently, we also found that the intake levels of the majority of these amine-related food and nutrition factors were not significantly different amongst the three HFS grades (Figure 5B). Taken together, these results suggested that dietary food and nutrition intake were possible contributing factors for the variation of the HFS-related amines in CRT; however, they were less likely to be the main contributing factors.

### Hand-Foot Syndrome-Related Transcriptome in Colorectal Tissue

**FIGURE 6:**
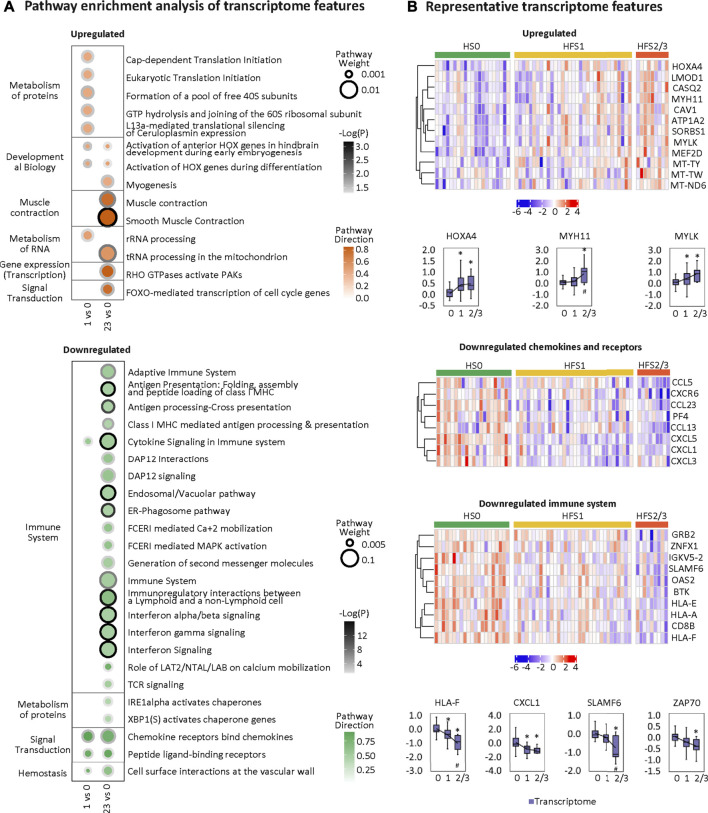
Transcriptome changes related to HFS susceptibility. **(A)** Pathway enrichment analysis of upregulated differentially expressed features (DEFs) (the upper left) and downregulated DEFs (the bottom left) on differential transcriptome. Cutoff: the minimum number of DEFs enriched in the pathway was set as 2, and the *p*-value was set as 0.05. The dot border color represents the –log10(*p*) of pathway significance. Pathway weight is defined as: (Enrichment ratio) × (sum of absolute value of DEF MOFA L1 weight in the pathway), and the dot size represents pathway weight. Pathway direction is the median log2 fold change (FC) of DEFs in the pathway (red, upregulated; green, downregulated). **(B)** Heatmaps (z-score) and boxplots show the expression profiles for representative transcriptome features for groups HFS0, HFS1, and HFS2/3. Statistical significance was set as: *p <  0.05 for HFS1 vs HFS0, HFS2/3 vs HFS0; ^#^ *p* < −0.05 for HFS2/3 vs HFS1 by differential expression analysis using limma.

Overall, the HFS-related upregulated transcriptome features were mainly enriched in pathways governing protein metabolism, development, muscle contraction, RNA metabolism, gene expression, and signal transduction. The most significantly altered pathways shared between HFS1 and HFS2/3 were two HOX-related pathways for development (Figure 6A). On the other hand, the downregulated transcriptome features were primarily enriched in pathways governing immunity, protein metabolism, signal transduction, and hemostasis. Interestingly, the number of pathways that were enriched by downregulated transcriptome features correlated positively with the severity of HFS. Genes with a high MOFA weight and connections to other omics data are presented in Figure 6B. In addition, CIBERSORT analysis based on the HFS-related transcriptome in CRT revealed that M0 macrophages were significantly negatively associated with HFS grade (**Additional file 2:** Supplementary Table S4). For macrophages M1 and M2, which are the downstream polarization product of M0, M1 demonstrated a negative trend with HFS grade, while M2 showed a positive trend with HFS grade.

### Hand-Foot Syndrome-Related DNA Methylation in Colorectal Tissue

**FIGURE 7:**
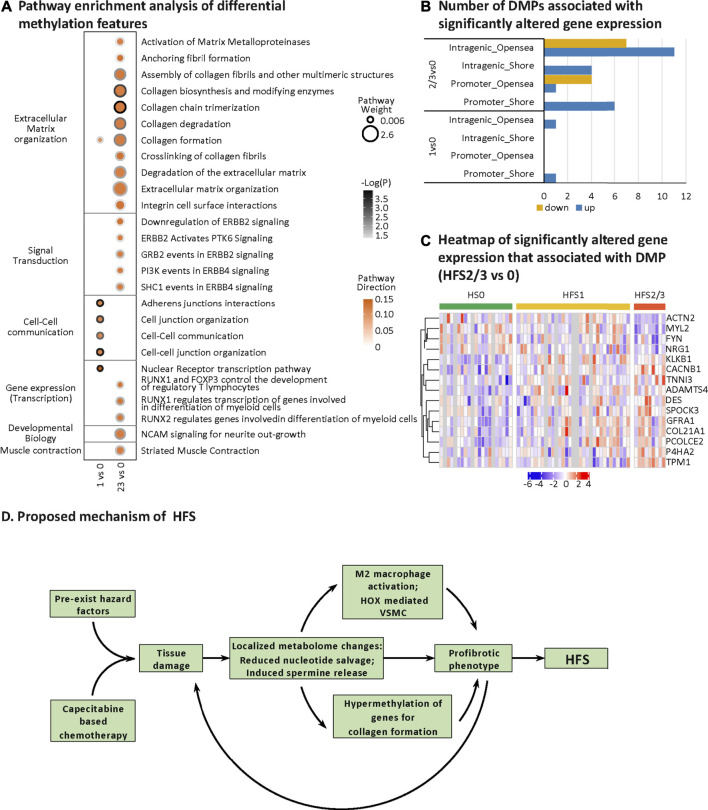
HFS-related DNA methylation changes and a proposed mechanism of chemotherapy-induced HFS. **(A)** Pathway enrichment analysis of differentially methylated genes (containing differentially methylated probes, DMPs). DMPs in opensea or shore regions were selected for the pathway enrichment analysis. Cutoff: the minimum number of DMPs enriched in the pathway was set as 5, and the *p*-value was set as 0.05. The dot border color represents the –log10(P) of pathway significance. Pathway weight is defined as: (Enrichment ratio) × (sum of absolute value of DMP MOFA LF1 weight in the pathway), and the dot size represents pathway weight. Pathway direction is the median Δβ of DMPs in the pathway (red, hypermethylated; green, hypomethylated). Notes: As for pathway direction, in the case of DMPs mapped to multiple regions (nine types of GDMR, gene-function-DMR: three gene-function, and three CpG-island-based regions), pathway direction is based on the direction of the region with the highest sum of absolute DMP MOFA L1 weight in the pathway. **(B, C)** Differential methylation–related transcriptional changes. Number of DMPs associated with significantly altered gene expression **(B)** and heatmap of significantly altered gene expression that associated with DMP (HFS 2/3 vs 0) **(C)**. **(D)** Proposed mechanism of HFS. Abbreviation: DMPs, differentially methylated probes. Notes: The nine types of GDMR were: 1) promoter-island regions, 2) promoter-shore/shelf regions, 3) promoter-opensea regions, 4) intragenic-island regions, 5) intragenic-shore/shelf regions, 6) intragenic-opensea regions, 7) intergenic-island regions, 8) intergenic-shore/shelf regions, and 9) intergenic-opensea regions.

HFS-related methylation changes were mainly enriched in the pathway for extracellular matrix (ECM) organization (Figure 7A). Collagen formation correlated positively with HFS susceptibility. In HFS2/3, more pathways within the ECM pathway were upregulated than in HFS1. A total of 15 genes that were differentially methylated were also differentially expressed in the CRT transcriptome (Figures 7B,C). These genes were hypermethylated in the CpG-shore and opensea regions of their promoter and intragenic regions, and most of these genes were upregulated in HFS samples.

### Potential Biomarkers for Hand-Foot Syndrome

**FIGURE 8:**
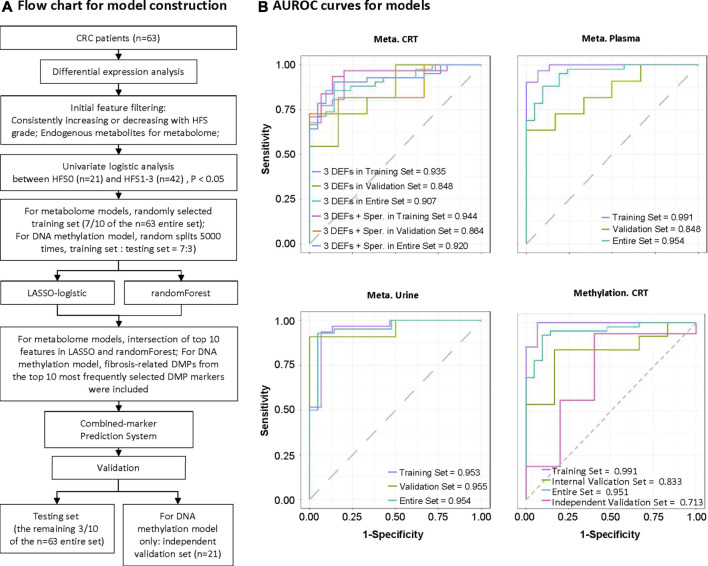
Construction of potential marker systems for HFS prediction. **(A)** Flow chart for model construction. The entire set was used to identify differentially expressed features (DEFs) in each omics dataset. During model development, we randomly divided the samples into training and validation sets (7:3). Models were developed to discriminate CRC patients who did not experience HFS (HFS0) from those with HFS (HFS:HFS1, HFS2/3). Models were constructed based on CRT metabolome, plasma metabolome, urine metabolome, and CRT methylation, respectively. **(B)** Respective ROC (receiver operating characteristic) curves for the developed models in the training set, validation set, and entire set. Note: Independent validation was performed for CRT methylation only. Abbreviation: Sper, spermine.

Multiple screening steps (Figure 8A) of our datasets (except for transcriptome data) yielded the following number of DEFs: 4 for CRT metabolome, 5 for plasma metabolome, 4 for urine metabolome, and **3** for DNA methylation data; these constituted potential biomarkers for HFS (**Additional file 2:** Supplementary Table S5). We further combined these features to construct a prediction model for each omics dataset (**Additional file 2:** Supplementary Table S6). All the prediction models showed reasonably good discrimination performance, with relatively high AUROC values ranging from 0.833 to 0.955 (Figure 8B). Other evaluation indices in the validation set also demonstrated relatively good predictive performance (Additional file 2: Supplementary Table S6). In addition, the AUROC for the DNA methylation model was 0.713 when examined by an independent testing dataset.

## Discussion

### Differences in Multi-Omics Profiles Predict the Hand-Foot Syndrome Susceptibility

Our results constituted the first demonstration that variations in multi-omics profiles could predict the HFS susceptibility (Figures 1D,E, 2). This was consistent with our previous report that variations in the endogenous urine metabolome correlated positively with HFS (Deng et al., 2020). As the CRT metabolome bears more similarities to that of skin than of plasma and urine in terms of their chemical composition, histology pattern, and cell developmental programs, changes in the CRT metabolome afford the best correlation with the HFS susceptibility.

Since HFS does not occur in CRT, and CRT had more HFS-related metabolome changes compared with plasma and urine, the HFS-related metabolomic alteration in CRT was less likely to be caused by diffusion or transportation from the skin through plasma to CRT. The more possible scenario was that the hazard factors inducing the susceptibility to HFS could also trigger a profound metabolic change in CRT. In another word, the HFS-related metabolic changes in CRT may infer the biomolecular mechanism of HFS susceptibility. The HFS-related changes in the CRT metabolome may provide clues to the biomolecular mechanism of HFS. Consistently, the barrier surface tissues, including the gut and skin, share similar mechanisms in regulating neuro-immune response (Veiga-Fernandes and Mucida, 2016) to potential hazards (Lou et al., 2016).

### Hand-Foot Syndrome-Related Alterations in the Metabolomes of Colorectal Tissue, Plasma, and Urine Indicate Potential Chronic Tissue Damage

The reduced metabolite levels in pathways (metabolism of nucleotides, nucleotide salvage, and nucleotide catabolism) in CRT and plasma of CRC patients indicated an imbalance or depletion of the nucleotide pool (Figures 3A,B,D), which can cause DNA replication stress (Forrer Charlier and Martins, 2020). These factors can cause damage to cells such as neurons that regenerate continuously (Fasullo and Endres, 2015), and cells that mediate the neuro-immune response. These cells are one of the main cell types of the barrier tissues such as skin and gut. Consistently, capecitabine-based chemotherapy can induce chronic peripheral-nerve damage and hence cause increased sensitivity to pain sensation (Banach et al., 2018). In response to inflammatory stimuli, injured cells release spermine into the extracellular space, which is a sign of upregulated tissue repair along with the consequent increase in cell proliferation (Zhang et al., 1999).

Consistent with the signs of HFS-associated damages to CRT, the HFS-related plasma (Figures 3B,D,E) and urine (Figures 3C,H) metabolomes were also characterized with signs of elevated cytotoxicity. In plasma, DPA is an essential omega-3 fatty acid, which is a component of phospholipids found in all animal cell membranes. DPA deficiency can lead to lesions of skin and connective tissues, causing atherosclerosis, coronary thrombosis, and multiple sclerosis (Kaur et al., 2011). DPA is a precursor of anti-inflammatory mediators, and it can also inhibit *COX2* activity. Choline and deoxycholic acid are bile acids that facilitate the uptake of dietary fats and excretion of cholesterol. Chronic exposure to elevated levels of bile acids can induce the generation of reactive oxygen species and reactive nitrogen species, resulting in damages to cellular membrane and DNA (Ghonem et al., 2015). In urine, salicylic acid is a cytotoxic microbial metabolite (Madan and Levitt, 2014). We found that the levels of both salicylic acid and its conjugation product correlated positively with HFS susceptibility. Pyroglutamic acid, which is found in substantial amounts in the skin, can act as another metabotoxin that causes acidosis (Emmett, 2014). L-Dopa is the precursor of dopamine, the accelerated excretion of which through urine is an indicator of potential neuronal damage (Lane, 2019).

Overall, our observed changes in metabolome datasets indicate that patients who are susceptible to HFS are likely to suffer from chronic tissue damage. We expect that this damage probably exists in their hands as well, contributing to HFS susceptibility. However, we could not identify the origin of this damage yet. Therefore, we focused on discussing the HFS-related metabolites from the enriched pathway with the highest MOFA weight, namely, spermine, serotonin, and spermidine, which were engaged in the organic cation transportation pathway. We then moved on to discuss the multi-omics changes that might be caused by these damages. We believe that features with a high MOFA weight can point to the most fundamental biomolecular changes of HFS susceptibility. The potential molecular pathological mechanism of HFS development can be refined by carefully interpreting these biomolecular changes in the background of the current understanding of these biomolecules.

### Chronic Tissue Damage Increases the Cellular Level of Spermine Which can Alter Immune Function and the Cellular DNA Methylation Profile

The elevated level of spermine that we observed in HFS susceptible patients was a consequence of leakage from damaged tissues as well as upregulated spermine biosynthesis as the damage persisted. The *de novo* biosynthesis of spermine and spermidine was not upregulated in the HFS1 group (Figure 4C). Ongoing tissue damage requires additional spermine, and therefore the HFS2/3 group exhibited increased *de novo* biosynthesis of both spermine and spermidine (Figure 4C). Was this caused by increased uptakes of these two metabolites? In healthy adults, polyamines are mainly derived from foods or are synthesized by the intestinal microbiota. It is known that polyamines in the intestinal tract are absorbed quickly, thereby rapidly increasing their levels in the portal vein, after which they are distributed to all organs and tissues (Soda, 2018). Based on our data for dietary intake of study participants, spermine level correlated positively with high intakes of manufactured meat and seafood; however, no significant association between excessive intake of either product type and HFS susceptibility was found (Figure 5B). Therefore, dietary habits were not the main reason for the observed differences in spermine levels among the various HFS groups.

Upregulated spermine can lead to a series of metabolic changes which were associated with substantial changes in the transcriptome. Spermine itself can be converted to spermidine (Hesterberg et al., 2018). Because the expression of the rate-limiting enzyme in this conversion (*SAT1*) did not correlate with HFS susceptibility (**Additional file 2:** Supplementary Table S3), increased spermidine level may be a consequence of increased spermine level. Spermine also stimulated the release of serotonin from mast cell granules (Figure 4D), which was in line with its function (Kanerva et al., 2009; García-Faroldi et al., 2010). All these amine molecules (spermine, spermidine, and serotonin) themselves serve as important immune function regulators in activating M2 macrophage polarization (Coates et al., 2017; Latour et al., 2020).

Spermine can also induce DNA methylation by suppressing the expression of *AMD1* (Soda, 2018). Since the substrate of *AMD1* is SAM, its level may be elevated subsequently. SAM is the methyl-donor for the DNA methyltransferase *DNMT1*. DNA methylation is controlled by the relative expression of genes encoding pro-methylation and demethylation enzymes (Greenberg and Bourc’his, 2019). Because the transcriptional expression of *DNMT1* and most of the other genes controlling DNA methylation correlated negatively with HFS susceptibility (Figure 4C and **Additional file 2:** Supplementary Table S7), the observed upregulation of DNA methylation could be mainly ascribed to an increase in available SAM brought about by suppression of *AMD1* expression. On the other hand, since the elevated supply of methionine and consumption co-factors ADP as the HFS grade increased, plus MAT had a trend of increase in HFS2/3, it was likely that the methionine cycle also contributed to an increased SAM level. This process might contribute more to the overall DNA hypermethylation profile.

### Variations in the Colorectal Tissue Transcriptome Correlate With Relative Proliferation or Suppression of Immune Cells

In line with the well-established function of spermine (Latour et al., 2020), the overall downregulation of immune function and induction of some immune-cell proliferation in patients with HFS was noticed. Depending on environmental factors, macrophages generally can be stimulated to develop into either pro-inflammatory M1 or M2 macrophages (Murray and Wynn, 2011). M2 macrophages with the immunosuppressive function are involved in tissue repair. Spermine suppresses the polarization of M1 macrophages yet activates the polarization of M2 macrophages.

Upregulated genes in CRT of HFS susceptible patients (HFS1 or HFS2/3 groups) indicated an induced proliferation phenotype. *HOXA4* belongs to the HOX (homeobox) family of transcription factors (Li et al., 2020). Serotonin plays multiple important roles in the immune system such as the activation of M2 macrophages (Wu et al., 2019). Consistently, *HOXA4* also induces the IL-6/STAT3 signaling pathway (Yang et al., 2011), which further activates M2 macrophage polarization (Yin et al., 2018). M2 macrophages are profibrotic, and their abundance are increased in the skin and plasma of patients afflicted with systemic sclerosis (Higashi-Kuwata et al., 2010). *HOXA4* deficiency is related to the downregulation of muscle contraction-related genes such as *MYH11* that are expressed only in smooth muscle (Kimura et al., 2020). Elevated levels of *HOXA4* may activate the expression of the genes encoding *MYH11* (Gomez and Owens, 2012) and *MYLK* (Yang et al., 2016), which are critical in transforming cells from a contractile phenotype to a proliferative state for ECM production (Kimura et al., 2020). Elevated ECM production is a risk factor for developing fibrosis-related tissue damage (Karsdal et al., 2017; Asano, 2018; Weiskirchen et al., 2019).

Downregulated genes in CRT of HFS susceptible patients (HFS1 or HFS2/3 groups) indicated an overall suppressed immune response. HLA-F can bind to ILT2, ILT4, resulting in the presentation of antigens to the T cells; the HLA-F/ILT2/ILT4 trimer also serves as a ligand for receptors expressed on natural killer (NK) cells (Lin and Yan, 2019). In parallel, downregulated *IL-17A* (**Additional file 2:** Supplementary Table S8) can reduce *CXCL1* expression (Figure 6B), thereby preventing neutrophils recruitment (Furue et al., 2020). *CXCL1* is a secreted growth factor that signals through the G-protein coupled receptor, CXC receptor 2. In HFS2/3, the decreased levels of immune cytokines caused profound suppression of the immune system (Figure 6A). Consistently, downregulated *SLAMF6* suppresses NK-cell activation (Ma et al., 2007), and downregulated *ZAP70* suppresses TCR signaling response (Gaud et al., 2018). *SLAMF6* belongs to the CD2 subfamily of the immunoglobulin superfamily, which is expressed on NK, T, and B lymphocytes. *ZAP70* belongs to the protein tyrosine kinase family, and it plays roles in T-cell development and lymphocyte activation. The overall suppressed immune response was not expected, as the most widely accepted mechanism of HFS is the *COX2* overexpression-mediated inflammation (Lou et al., 2016). We did find evidence suggesting that *COX2* was activated, such as the reduced level of DPA in plasma (DPA is a potent *COX2* inhibitor) (Kaur et al., 2011) and the elevated level of prostaglandin F3α (PGF3α). In our study, *COX2* was not expressed in CRT (**Additional file 2:** Supplementary Table S9). Therefore, HFS treatments that focus on the suppression of the immune response may not have the expected benefits.

### Hand-Foot Syndrome-Related DNA Methylation in Colorectal Tissue Favors a Profibrotic Phenotype

The human genome contains 42 different collagen genes for 28 different types of collagens. Here we found that 25 collagen genes for 20 types of collagens were all hypermethylated in CRT of patients susceptible to HFS (Karsdal et al., 2017). It is noteworthy that, in our study, changes in the expression of genes that govern the methylation pattern did not correlate well with the overall DNA hypermethylation. Therefore, other mechanisms may account for the observed dynamics of DNA methylation in response to the increased spermine level and the consequent hypermethylation of targeted genes. Our results support the notion that the effects of DMPs on gene expression are dependent on functional gene regions (gene body, promoter), and intergenic regions (Greenberg and Bourc’his, 2019) as well as the relative distance between CpG islands (Klett et al., 2018). The hypermethylated promoter-shore and intragenic opensea regions may elicit positive effects on gene expression. The upregulated collagen genes, such as *PCOLCE2* (Ulmasov et al., 2013), *ADAMST4* (Lu et al., 2018), *DES* (Torricelli et al., 2016), and *TPM1* (Huang et al., 2020), participate in ECM organization and collagen formation. Prolonged remodeling of the ECM and collagen formation can further lead to a profibrotic phenotype—and even fibrosis-related tissue damage (Asano, 2018).

### Proposed Mechanism for the Chemotherapy-Induced Hand-Foot Syndrome

On the basis of our multi-omics data and published information on HFS, we proposed a mechanism for chemotherapy-induced HFS (Figure 7D). Specifically, damage to tissues, including those harboring neurons and mast cells with essential neuro-immune functions, can initially cause localized changes to the metabolome including a decrease in the nucleotide pool and an increase in spermine release. An acceleration of changes to the metabolome may lead to a profibrotic phenotype characterized by the suppressed immune function, elevated cellular proliferation, and tissue fibrosis. Once Capecitabine-based chemotherapy is administrated, it adds to the existing hazard factors and causes severe tissue damage (Tsai et al., 2020). Finally, the accumulated molecular changes lead to severe cellular proliferation and fibrosis of tissues, and ultimately to HFS.

The resident profibrotic cells in organs can develop to myofibroblasts, which are the key players in the damage progression related to fibrosis. The resident profibrotic cells include epithelial cells from the skin and CRT (Weiskirchen et al., 2019). Fibrosis is also the main characteristic of systemic sclerosis, damaging the skin and various internal organs (Asano, 2018). This partially explained why the multi-omics variation in CRT was closely related to HFS susceptibility. Based on the similarity between skin where the HFS happened and CRT from where the experimental tissue was acquired and screened, the HFS-related metabolome in CRT was more likely a reflection or an indicator of what was happening in the skin at a biomolecular level. With the current data and knowledge, we could not further tell how the HFS-related metabolome affects skin. However, spermine, which was recognized as the most important HFS-related metabolite in CRT, can cause overall DNA hypermethylation, especially on genes from collogen formation. Spermine can also cause overall downregulated genes related to M2 macrophage activation and suppressed immune response. These biomolecular changes were important signs of systematic fibrosis. Therefore, suppression of systematic fibrosis may provide an alternative treatment target in the prevention of HFS.

### Prediction Models for Chemotherapy-Induced Hand-Foot Syndrome

To assist clinical practice for CRC patients undergoing capecitabine chemotherapy, we additionally developed prediction models for HFS. Samples of cellular mRNA are relatively difficult to preserve, and the use of mRNA for routine screening is relatively costly; moreover, models using biomarkers from other omics data also show good enough predictive abilities. Therefore, we developed a set of prediction models only using metabolome and DNA methylation data (Figure 8 and **Additional file 2:** Supplementary Table S5 and Supplementary Table S6).

Considering the significance of spermine, we combined it with the three biomarkers, including N-octanoyl-d-sphingosine (C8-ceramide), O-phosphoethanolamine, adenylosuccinic acid into the CRT metabolome model, and a slight increase in AUROC in the validation set was shown (Figure 8B and **Additional file 2:** Supplementary Table S6). Both C8-ceramide and O-phosphoethanolamine are implicated in sphingolipid metabolism, the alternation of which plays different regulatory functions in multiple cell events. Ceramides serve as a localized cytokines milieu to regulate inflammatory function at barrier organs such as skin and their levels are directly related to disease severity (Toncic et al., 2020). Ceramides can also modulate the serotonin release from mast cells (Ji et al., 2011).

Adenylosuccinic acid participates in purine metabolism. In line with biomarkers in the CRT metabolome, most of the plasma metabolite biomarkers, including PC[14:1(9Z)/24:1(15Z)], PC(35:2), PC(34:2), and cis-8,11,14,17-eicosatetraenoic acid are lipid metabolism-related, which suggested that patients susceptible to HFS had a remarkable disturbed lipid metabolism. In addition, an elevated level of dodecanoic acid plays roles in fatty acid biosynthesis.

For the urine prediction model, 4 potential biomarkers, including 4-pyridoxolactone, PA(27:6), glycodeoxycholic acid, and 5,6-dihydrothymine generally reflected different alterations of VB6 metabolism, fatty acid and lipid metabolism, pyrimidine metabolism.

All of the three DNA methylation biomarkers were associated with the profibrotic phenotype. SMIM24 (previously termed C19orf77) is a member of the small integral membrane protein family, the downregulation of which is related to steatosis, glucose intolerance, inflammation, and fibrosis in high-fat diet-, high-fat-high-cholesterol diet-, and methionine-choline-deficient diet feed mice (Song et al., 2021). SMIM24 has an important paralog PDZK1, the downregulation of which can cause myocardial infarction-related fibrosis (Yesilaltay et al., 2009). On the other hand, MIR130A promotes collagen secretion, myofibroblast transformation through CYLD, enhancing Akt activity (Zhang et al., 2019). The level of MIR130A positively correlated with both skin and cardiac fibrosis (Li et al., 2017). At last, mutation in PLEKHA7 is negatively related to perivascular fibrosis in the heart and kidney (Endres et al., 2014).

The major strengths of this study include the first use of metabolome and DNA methylation markers to predict HFS susceptibility, various datasets, and the adjustments for potential confounding factors. However, our models also had limitations. First, the sample size was relatively small. Second, the prediction model for HFS2/3 patients is also of greater importance. Owing to the limited number of HFS2/3 patients who were enrolled in our study; however, we were unable to produce a specific prediction model for HFS2/3 alone. Therefore, further validation promises better applicable results.

## Conclusion

In summary, our results demonstrated that a multi-omics profibrotic phenotype was closely associated with chemotherapy-induced HFS. On top of this, the metabolome variation in CRT showed a tighter correlation with HFS susceptibility than in plasma and urine. The metabolome changes for each of matched plasma, urine, and CRT in relation to HFS were characterized by chronic tissue damage, which was indicated by reduced nucleotide salvage, elevated spermine release, and increased production of endogenous cytotoxic metabolites. HFS-related transcriptome changes of CRT showed an overall suppressed inflammation profile but increased M2 macrophage polarization. HFS-related DNA methylation of CRT presented gene-specific hypermethylation on genes mainly for collagen formation. The hypermethylation was accumulated in the opensea and shore regions, which elicited a positive effect on gene expression. Additionally, we developed and validated models combining multiple biomarkers to predict HFS, with reasonably good discrimination. Our findings provide novel insights into the susceptible factors contributing to HFS, and advance a better understanding of the molecular mechanism underlying HFS, which can promote the implementation of individualized treatment against HFS. Nonetheless, further studies based on large cohorts to verify our findings are warranted.

## Data Availability

The datasets presented in this study can be found in online repositories. The names of the repository/repositories and accession number(s) can be found below: https://www.ncbi.nlm.nih.gov/geo/, GSE171468; https://www.ncbi.nlm.nih.gov/geo/, GSE171550.

## Glossary

AMD1: adenosylmethionine decarboxylase 1
AUROC: area under the receiver operating characteristic curve
BMI: body mass index
C8-ceramide: N-octanoyl-d-sphingosine
CRAE: chemotherapy-related adverse event
CTCAE: Common Terminology Criteria for Adverse Events
CRC: colorectal cancer
CRT: normal colorectal tissue
DDC: dopa decarboxylase
DEF: differentially expressed feature
DHF: dihydrofolate
DMP: differentially methylated probe
DPA: docosapentaenoic acid
ECM: extracellular matrix
FC: fold of change
FFQ: food-frequency questionnaire
FPKM: fragments per kilobase of transcript per million fragments mapped
HFS: hand-foot syndrome
LASSO: least absolute shrinkage, and selection operator
LF: latent factor
MAT: methionine adenosyltransferase
MOFA: multi-omics factor analysis
NK: natural killer
ODC: ornithine decarboxylase
PCA: principal component analysis
PGF3α: prostaglandin F3α
PVCA: principal variance component analysis
SD: standard deviation
VB6: vitamin B6
XELOX: capecitabine plus oxaliplatin
