# Supplementary material for: Spermine-Related DNA Hypermethylation and Elevated Expression of Genes for Collagen Formation are Susceptible Factors for Chemotherapy-Induced Hand-Foot Syndrome in Chinese Colorectal Cancer Patients
Source: Front Pharmacol. 2021 Sep 1;12:746910. doi: 10.3389/fphar.2021.746910 (PMC8440935; doi:10.3389/fphar.2021.746910)
Supplement: Supplementary file 1 [file DataSheet1.docx]

Supplementary Material

**This DOCX file includes:**

Supplementary Methods

**Other supplementary materials for this manuscript includes:**

Additional file 2: Supplementary Tables S01 to S16 (data sheet 2.xlsx).

# Supplementary Methods

## Detailed methods for metabolome analyses

***Reagents, and sample preparation***

LC-MS (liquid chromatography-mass spectrometry)-grade water (H_2_O) and methanol were purchased from Honeywell (Muskegon, USA). LC-MS-grade acetonitrile was purchased from Merck (Darmstadt, Germany). Ammonium bicarbonate (NH_4_HCO_3_) and formic acid (HCOOH) were purchased from Sigma-Aldrich (St. Louis, USA). For urine and plasma samples (100 µL), 400 μL methanol was added containing all of the following internal standards: carnitine C2:0-d3, 0.16 µg/mL; carnitine C8:0-d3, 0.1 µg/mL; carnitine C10:0-d3, 0.1 µg/mL; carnitine C16:0-d3, 0.15 µg/mL; LPC 19:0, 0.75 µg/mL; FFA C16:0-d3, 2.5 µg/mL; FFA C18:0-d3, 2.5 µg/mL; CDCA-d4, 1.5 µg/mL; CA-d4, 1.85 µg/mL; Trp d5, 4.25 µg/mL; Phe-d5, 3.6 µg/mL; SM 12:0, 0.75 µg/mL; choline d4, 2.0 µg/mL. Samples were then mixed for 60 s, followed by centrifugation at 14,000 × *g* at 4°C for 15 min. Finally, each supernatant was transferred to an HPLC glass vial and stored at –20°C for subsequent LC-MS/MS analysis. For tissue samples, 75% methanol (8-fold of tissue, ~50 mg) was added to normal colorectal tissue (CRT) in a 2-mL tube, which was sonication at 66 Hz at 4°C for 4 min, followed by centrifugation at 14,000 × *g* at 4°C for 15 min. Finally, each supernatant was transferred to an HPLC glass vial and stored at –20°C for subsequent LC-MS/MS analysis. For each type of the metabolome sampling, an equivalent amount of each study sample was pooled together to produce quality control (QC) samples, and the QC samples were processed in the same way as the other study samples. One QC sample was injected during the screening batch at every ten study samples intervals to eliminate the systemic error.

***Data acquisition***

Metabolome data for samples of urine, plasma, and tissues from CRC patients were acquired using an UHPLC system (1290 series; Agilent Technologies, USA) coupled to a quadruple time-of-flight mass spectrometer (Q Exactive, Thermo Scientific, USA). A Waters ACQUITY UPLC BEH C8 column [particle size, 1.7 μm; 100 mm (length) × 2.1 mm (i.d.)] and Waters ACQUITY HSS T3 column [particle size, 1.8 μm; 100 mm (length) × 2.1 mm (i.d.)] were used for LC in positive electrospray ionization (ESI+) and negative (ESI–) modes, respectively, and each column was maintained at 50°C. For urine samples, mobile phase A was 0.1% HCOOH in water, and B was 0.1% HCOOH in acetonitrile for both ESI+ and ESI– modes. For plasma and tissue samples, mobile phase A was 0.1% HCOOH in water, and B was 0.1% HCOOH in acetonitrile for ESI+ mode; for ESI– mode, mobile phase A was 6.5 mM NH_4_HCO_3_ in water, and B was 6.5 mM NH_4_HCO_3_ in 95% methanol. The flow rate was 0.3 mL/min. Gradient elution was carried out as follows: for ESI+ mode, 0–1 min: 5% B, 1–24 min: 5–100% B, 24.1–27.5 min: 100% (B), 27.6–30 min: 100–5% (B) for re-equilibration. For ESI– mode, the gradient was: 0–1 min: 5% B, 1–18 min: 5–100% B, 18.1–22 min: 100% (B), 22.1–25 min: 100–5% (B) for re-equilibration. The injection volume was 10 μL. All samples were randomly injected during data acquisition.

***Data processing***

Nontargeted LC−MS data from multiple runs were extracted and aligned using XCMS based on one-step metabolomics (One-Map) platform (http://www.5omics.com/). The key parameters for plasma and urine were set as follows: method = “centWave”; ppm = 15; snthr = 10; peakwidth = c(5, 40); minifrac = 0.5, and the key parameters for tissue were set as follows: method = “centWave”; ppm = 20; snthr = 4; peakwidth = c(5, 30); prefilter = c(3, 15000). The generated MS^1^ peak table includes the mass-to-charge ratio (m/z), RT, peak abundances, and other information. For each detected metabolite, the generated theoretical m/z, RT, and surrogate MS^2^ spectrum were matched to the experimental MS^1^ m/z (tolerance: ±0.01Da), and MS^2^ spectrum (tolerance: ±0.05Da). The metabolites were identified according to KEGG database (https://www.genome.jp/kegg/) and an Integrated database (One-Map platform), with RT tolerance of ±18s and ±24s, respectively. The normalization procedures for the three metabolome data sets were completed by using the online tool Metaboanalyst (<https://www.metaboanalyst.ca>, version 4.0).
